# Characterisation of Antiviral Activity of Cathelicidins from Naked Mole Rat and *Python bivittatus* on Human Herpes Simplex Virus 1

**DOI:** 10.3390/ph14080715

**Published:** 2021-07-24

**Authors:** Alexia Damour, Magali Garcia, Hye-Sun Cho, Andy Larivière, Nicolas Lévêque, Chankyu Park, Charles Bodet

**Affiliations:** 1 Laboratoire Inflammation Tissus Epithéliaux et Cytokines (LITEC EA 4331), Université de Poitiers, CEDEX 9, 86073 Poitiers, France; alexia.damour@univ-poitiers.fr (A.D.); Magali.GARCIA@chu-poitiers.fr (M.G.); Andy.LARIVIERE@chu-poitiers.fr (A.L.); Nicolas.LEVEQUE@chu-poitiers.fr (N.L.); 2 Laboratoire de Virologie et Mycobactériologie, CHU de Poitiers, 86021 Poitiers, France; 3 Department of Stem Cell and Regenerative Biotechnology, Konkuk University, Seoul 143-701, Korea; chssky77@gmail.com (H.-S.C.); chankyu@konkuk.ac.kr (C.P.)

**Keywords:** Hg-CATH, Pb-CATH4, herpes simplex virus I, keratinocytes, antiviral

## Abstract

Hg-CATH and Pb-CATH4 are cathelicidins from *Heterocephalus glaber* and *Python bivittatus* that have been previously identified as potent antibacterial peptides. However, their antiviral properties were not previously investigated. In this study, their activity against the herpes simplex virus (HSV)-1 was evaluated during primary human keratinocyte infection. Both of them significantly reduced HSV-1 DNA replication and production of infectious viral particles in keratinocytes at noncytotoxic concentrations, with the stronger activity of Pb-CATH4. These peptides did not show direct virucidal activity and did not exhibit significant immunomodulatory properties, except for Pb-CATH4, which exerted a moderate proinflammatory action. All in all, our results suggest that Hg-CATH and Pb-CATH4 could be potent candidates for the development of new therapies against HSV-1.

## 1. Introduction

Human herpes simplex viruses (HSV)-1 and -2 are enveloped DNA viruses that belong to the *Herpesviridae* family and *Alphaherpesvirinae* subfamily, which also includes varicella-zoster virus (VZV) [1,2]. More than one-third of the world population is exposed to HSV and transmission occurs through direct contact with infected secretions [3]. HSV is a neurotropic and dermotropic virus involved in skin and mucosa infections by replicating in stratified squamous epithelia where keratinocytes are the first cell target. This virus is also responsible for severe neonatal and neurological infections. HSV-1 used to be mainly responsible for oral, ocular, and neurological infections, while HSV-2 caused genital and neonatal infections, but because of changes in oral sex practices over recent years, HSV-1 is now also involved in genital infections [4,5,6,7,8]. Classically, skin and mucosal infections are localised, but disseminated and potentially life-threatening infections can occur as eczema herpeticum or neonatal infections when the skin barrier or patient immune system are compromised [9].

Currently, nucleoside analogue drugs such as acyclovir (ACV) used for the treatment of HSV infections interfere with viral replication without altering the infectivity of existing viral particles. However, ACV can be responsible for renal cytotoxicity with acute renal failure requiring dialysis [10]. Moreover, resistance against these historical treatments has been emerging, especially in immunocompromised patients, and is related to mutations of the viral thymidine kinase (TK) or DNA polymerase genes [11,12]. Consequently, the development of new antiviral molecules exerting a strong antiviral activity on both HSV-1 replication and infectious viral particles represents a major challenge.

Antimicrobial peptides (AMPs) are classically small, cationic peptides, ranging from 12 to 50 amino acids, and are part of the innate immune response against pathogens [13]. According to the AMP database, more than 2600 AMPs have been identified in microorganisms, plants, vertebrates, and invertebrates, and some of them showed broad-spectrum antimicrobial activity [14], which can be associated with microbicidal properties and/or immunomodulatory effects on the host response [15,16,17]. Among AMP families, cathelicidins have demonstrated potent antimicrobial activity against fungi, parasites, Gram-positive and Gram-negative bacteria, and viruses [16,17]. Cathelicidins are characterised by two functional domains, the conserved cathelin-like proregion, and the N-terminal active domain region, [15,16,18]. Cathelicidins exhibit antiviral properties by directly interacting with the viral envelope or by indirect activity through immunomodulation of the host response [17]. Recently, the human cathelicidin LL-37 has been shown to significantly reduce HSV-1 replication in keratinocytes through immunomodulation of the host antiviral response involving the interferon (IFN) pathway [19]. Thus, AMPs from the cathelicidin family could represent an interesting alternative to classical anti-HSV-1 treatments as regards their previously described antiviral effect.

In this work, we evaluated the antiviral activity of two cathelicidins recently identified in animals, Hg-CATH and Pb-CATH4. Hg-CATH is the only identified cathelicidin from the naked mole rat *Heterocephalus glaber*, a long-lived rodent known to be resistant to spontaneous and experimentally induced cancers [20]. This AMP exhibits a strong bactericidal effect against Gram-negative bacteria such as *Escherichia coli*, *Pseudomonas aeruginosa*, and *Salmonella typhimurium* including antibiotic-resistant clinical strains [21]. Pb-CATH4, isolated from the *Python bivittatus*, has potent activity against both Gram-negative bacteria, including multiresistant clinical strains, and Gram-positive bacteria such as *Bacillus cereus* [22]. Hg-CATH and Pb-CATH4 seem to act by disrupting the bacterial membrane following AMP binding. In addition, both of them have good serum stability and exert low cytotoxicity on various mammalian cell types, making them excellent candidates for therapeutic applications [21,22].

## 2. Results

### 2.1. Comparison of Amino Acid Sequences and Peptide Structures of Hg-CATH and Pb-CATH4

**Figure 1:**
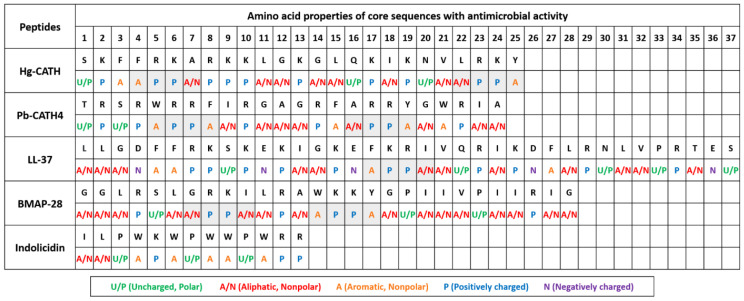
Comparison of the amino acid sequence and properties of five AMPs with anti-HSV-1 activity. Amino acid sequences of cathelicidins and chemical properties in different colours corresponding to each amino acid were shown. The definition of chemical properties was shown at the bottom. Numbers at the top indicate the position of amino acids. The conserved motif, A(A/N)-P-P-A(A/N), was highlighted in grey.

The active core region sequences of Hg-CATH and Pb-CATH4 consist of 25 and 24 amino acids, respectively. Amino acid sequence homology between the two peptides is 20%, and they have several common biochemical characteristics (Table S1) [21,22]. The results of structural predictions for each peptide were consistent between secondary and tertiary structures regarding the constitution of helical and coil regions (Figures S1 and S2A). While both peptides form alpha-helical structures, the length of helical and coil regions in each peptide was different (Figures S1 and S2A). In addition, Hg-CATH is rich in lysine with some arginine, possessing the cationic nature of cathelicidins, while only arginine was present for the positivity in Pb-CATH4. Compared to other cathelicidins with anti-HSV-1 activity, including the bovine myeloid antimicrobial peptide called BMAP-28, indolicidin, and LL-37 [19,23,24,25], the biochemical characteristics of both Hg-CATH and Pb-CATH4, especially high net charge and alpha helicity, are close to those of LL-37 (Figure S2B). Interestingly, the sequence analysis of all these peptides revealed the presence of the A(A/N)–P–P–A(A/N) motif (A, A/N, and P for aromatic, aliphatic/nonpolar, and positively charged amino acids, respectively) at least once in their sequence, except for indolicidin (Figure 1). No information is currently available for this A(A/N)–P–P–A(A/N) motif; we hypothesise that its presence could be related to the antiviral properties of peptides.

### 2.2. Cytotoxicity of Hg-CATH and Pb-CATH4 on Primary Human Keratinocytes

**Figure 2:**
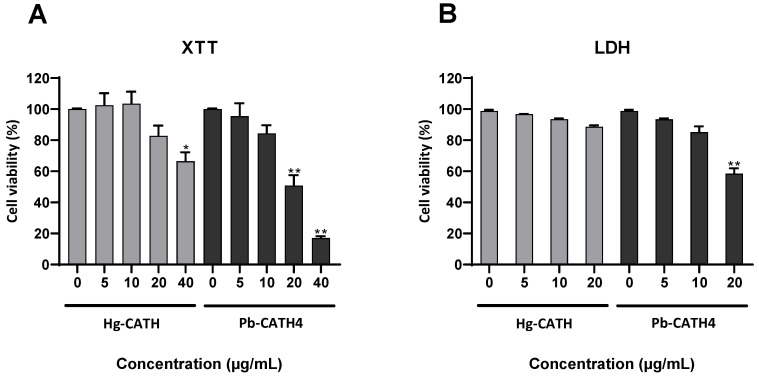
Evaluation of Hg-CATH and Pb-CATH4 cytotoxicity on primary human keratinocytes. Keratinocytes were exposed to Hg-CATH or Pb-CATH4 for 24 h. Cell viability was evaluated using the XTT cell viability assay (**A**) or LDH assay (**B**). Data are represented as mean + standard error of mean (SEM) of four independent experiments. * *p* < 0.05, ** *p* < 0.01 compared with the untreated control.

The cytotoxicity of 5, 10, 20, and 40 µg/mL of Hg-CATH or Pb-CATH4 was evaluated on primary human keratinocytes, after 24 h of incubation, using XTT and LDH assays (Figure 2). Cell viability at a final concentration of 40 µg/mL for Hg-CATH or from 20 µg/mL for Pb-CATH4 was significantly decreased, whereas no significant cytotoxicity was noted at the lower concentrations. The following experiments were performed using the noncytotoxic concentrations of 10 or 20 µg/mL for Hg-CATH and 10 µg/mL for Pb-CATH4.

### 2.3. Hg-CATH and Pb-CATH4 Reduce HSV-1 Replication in Primary Human Keratinocytes

**Figure 3:**
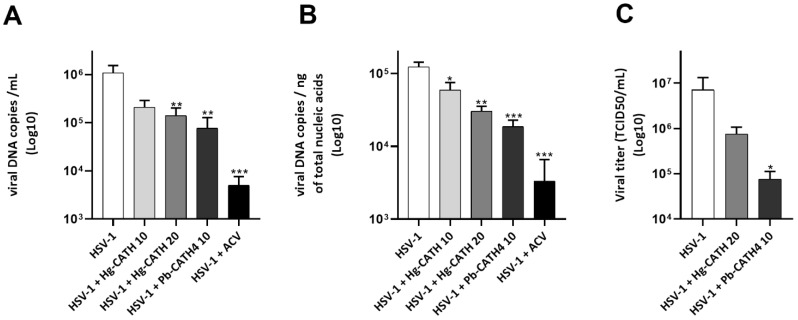
Evaluation of Hg-CATH and Pb-CATH4 antiviral activities during primary human keratinocyte infection with HSV-1. Primary human keratinocytes were treated with 10 or 20 µg/mL of Hg-CATH, 10 µg/mL of Pb-CATH4, or 10 µg/mL of ACV before being infected 24 h with HSV-1 at a multiplicity of infection (MOI) of 0.1. Viral DNA quantification was determined in cell supernatant ((**A**), log10 viral DNA copies/mL) and in cell monolayer ((**B**), log 10 viral DNA copies/ng of total nucleic acids) from infected keratinocytes. Viral titre in cell culture supernatant was determined by end-point dilution assay and expressed in TCID50/mL (**C**). Data are represented as mean + standard error of mean (SEM) of five independent experiments. * *p* < 0.05, ** *p* < 0.01, *** *p* < 0.001 compared with the infected control without AMPs.

In order to assess the antiviral effect of cathelicidins on HSV-1 replication in primary human keratinocytes, cells were incubated for 1 h with Hg-CATH, Pb-CATH4, or ACV prior infection with HSV-1 at an MOI of 0.1 for 24 h. The viral load in cell culture supernatants of primary human keratinocytes treated with 20 µg/mL of Hg-CATH or 10 µg/mL of Pb-CATH4 was significantly reduced, by about 1 log (87% and 93% reduction, respectively), compared to untreated cells (Figure 3A). Viral replication was also significantly reduced in cell monolayers of treated cells. This effect was concentration dependent for Hg-CATH, 10 and 20 µg/mL inducing, 52% and 75% reduction, respectively, of viral DNA copy number. The adjunction of 10 µg/mL of Pb-CATH4 resulted in a greater antiviral effect, reducing by 85% the HSV-1 viral load in keratinocyte monolayer (Figure 3B). Otherwise, ACV, used as a positive control, reduced viral load by 2.6 log (99.5% reduction) in cell culture supernatants and by 1.6 log (97% reduction) in cell monolayers at a final concentration of 10 µg/mL. In addition, the antiviral effect of Hg-CATH and Pb-CATH4 in cell supernatant was checked by determining HSV-1 titre in cell culture supernatants. Treatment of primary human keratinocytes with 20 µg/mL of Hg-CATH or 10 µg/mL of Pb-CATH4 reduced the production of infectious viral particles by 89% and 99%, respectively (Figure 3C). All in all, Hg-CATH and Pb-CATH4 significantly decreased viral replication and infectious viral particle production in primary human keratinocytes.

### 2.4. Hg-CATH and Pb-CATH4 Have No Virucidal Activity against HSV-1

**Figure 4:**
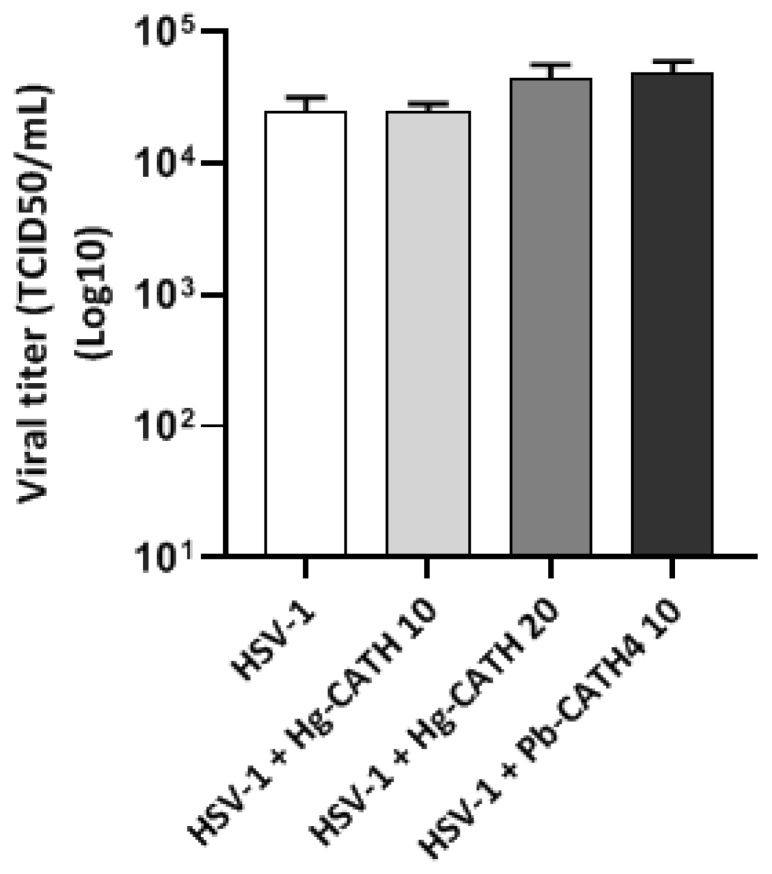
Evaluation of virucidal effect of Hg-CATH and Pb-CATH4. HSV-1 suspension was pre-incubated in absence or presence of Hg-CATH (10 and 20 µg/mL) and Pb-CATH4 (10 µg/mL) for 1 h at 37 °C before titration by endpoint dilution assay on Vero cells. Viral titres are expressed in TCID50/mL. Data are represented as mean + SEM of three independent experiments.

HSV-1 was incubated 1 h at 37 °C with or without Hg-CATH and Pb-CATH4 at, respectively 10 or 20 µg/mL and 10 µg/mL before measuring the residual infectious titre. No difference in the infectious titre of viral suspensions treated or not treated with peptides was observed, a finding suggesting that Hg-CATH and Pb-CATH4 are not virucidal to HSV-1 (Figure 4).

### 2.5. Modulation of the Innate Immune Response of Keratinocytes by Hg-CATH and Pb-CATH4

**Figure 5:**
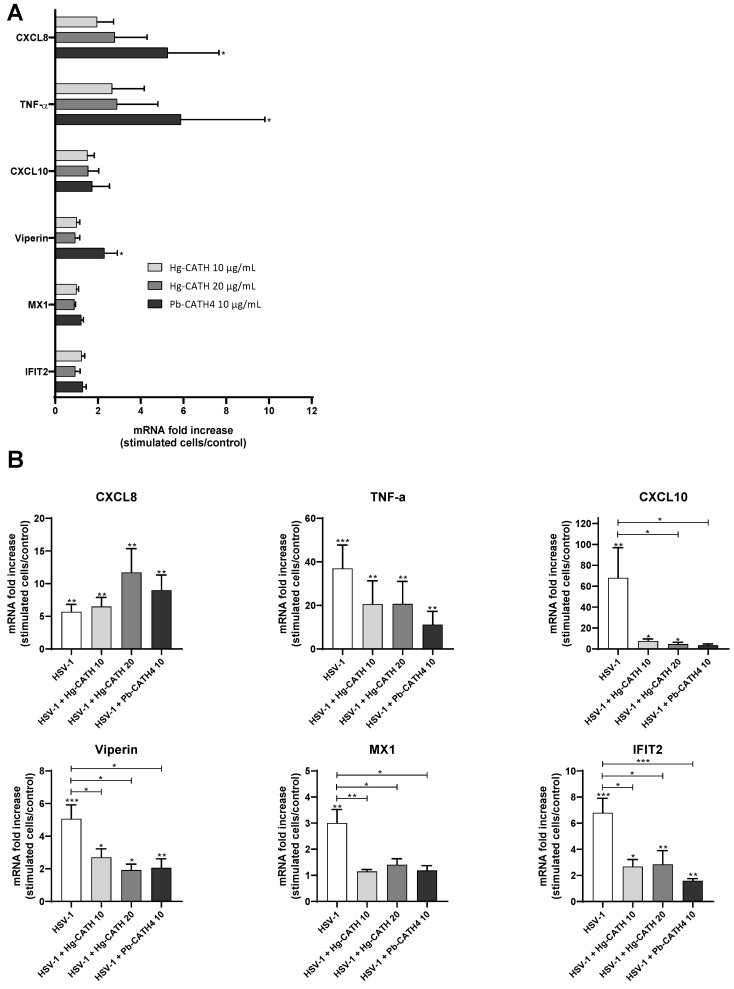
Evaluation of immunomodulatory properties of Hg-CATH and Pb-CATH4. CXCL8, TNFα, CXCL10, Viperin, MX1, and IFIT2 mRNA expression has been measured in keratinocytes stimulated by Hg-CATH (10 or 20 µg/mL) or Pb-CATH4 (10 µg/mL) (**A**). This expression has been then measured in keratinocytes stimulated with peptides at the same concentrations, and infected with HSV-1 at an MOI of 0.1 or mock-infected for 24 h (**B**). Data are represented as mean + SEM five independent experiments. * *p* < 0.05, ** *p* < 0.01, *** *p* < 0.001.

Inflammatory gene expression in primary human keratinocytes infected with HSV-1 or mock-infected in the presence or absence of Hg-CATH and Pb-CATH4 was assessed by RT-qPCR. The panel of genes studied included the chemokines CXC motif ligand (CXCL)8 known to be a powerful neutrophil chemoattractant protein, the cytokine tumour necrosis factor (TNF)-α, a strong regulator of innate immunity and inflammation through the induction of chemokine and cytokine expression, and the interferon-stimulated genes (ISGs) Viperin, interferon-induced protein with tetratricopeptide repeats 2 (IFIT2), CXCL10 and MX dynamin-like GTPase 1 (MX1) known for their antiviral properties. Firstly, the proinflammatory properties of Hg-CATH and Pb-CATH4 on uninfected keratinocytes were studied (Figure 5A). While Hg-CATH did not significantly modulate the mRNA level of all the genes studied, Pb-CATH4 increased CXCL8, TNF-α, and Viperin mRNA level at 24 h poststimulation (Figure 5A). Thus, Pb-CATH4 seems to exert a moderate proinflammatory effect on primary human keratinocytes. During keratinocyte infection with HSV-1, both Hg-CATH and Pb-CATH4 induced CXCL8 mRNA expression compared to untreated keratinocytes (Figure 5B). In contrast, mRNA expression of CXCL10, Viperin, MX1, and IFIT2 was significantly reduced (Figure 5B). Our results showed that Hg-CATH and Pb-CATH4 did not stimulate the antiviral defenses of primary human keratinocytes during HSV-1 infection.

## 3. Discussion

The aim of this study was to evaluate the antiviral activity of cathelicidins recently discovered in the naked mole rat (Hg-CATH) and the *Python bivittatus* (Pb-CATH4) against HSV-1. In accordance with the pathophysiology of the infection, this evaluation was carried out during the infection of human primary keratinocytes.

In our model, keratinocytes were first treated by peptides for one hour prior to a 24 h infection in the presence of the peptides. Hg-CATH or Pb-CATH4 demonstrated a strong ability to reduce HSV-1 replication since the viral load was significantly decreased in both the cells and the supernatants. In addition, these peptides reduced by up to 99% the production of infectious viral particles by infected cells measured by titration of cell culture supernatants on Vero cells. Previously, other cathelicidins have been previously identified as potent antiviral AMPs. Indeed, the human cathelicidin LL-37 has demonstrated antiviral effects against a broad spectrum of naked and enveloped viruses such as VZV, HSV-1, HSV-2, human herpesvirus 8, dengue virus, Zika virus (ZIKV), human papillomaviruses, and vaccinia virus [17]. Other animal cathelicidins have also been identified as potential candidates for antiviral therapies. This is the case for the chicken Cathelicidin B1, which inhibited the Influenza A virus (IAV) infection by blocking viral entry [26]. ModoCath5, a cathelicidin from the grey short-tailed opossum, has exhibited antiviral activity against West Nile virus, while the bovine cathelicidin BMAP-18 reduced ZIKV replication in human primary keratinocytes and in Vero cells, respectively [27,28]. Finally, a cathelicidin isolated from the venom of the *Bungarus fasciatus* snake has been reported to possess strong antiviral effects on IAV and ZIKV [29,30]. However, thus far, only the human LL-37, the bovine BMAP-28, and the indolicidin have been identified as inhibitors of HSV-1 replication [19,24,25]. Our results demonstrated the anti-HSV-1 properties of two new cathelicidins.

In order to identify the mechanism of action of these two AMPs against HSV-1, experiments were performed to assess whether Hg-CATH and Pb-CATH4 exerted a virucidal effect. Preincubation of each peptide with HSV-1 did not reduce HSV-1 titre, a finding that suggests Hg-CATH and Pb-CATH4 were not able to induce alteration of viral particles.

However, the absence of virucidal activity does not exclude the capacity of these peptides to directly inhibit one of the stages of the virus replication cycle. Indeed, another hypothesis may be to consider an inhibition of virus attachment to its receptors, namely, herpes virus entry mediator (HVEM), nectin-1, nectin-2, and heparan sulphate at the keratinocyte surface [7,31] This was reported for human β-defensin 3, which bound to both HSV glycoprotein B and host cells heparan sulphate leading to inhibition of virus attachment and internalisation into host cells [32] In the same way, LL-37 could block HSV-1 binding to corneal cells, preventing virus entry and spreading [33]. Moreover, AMPs can interfere with intracellular steps of viral replication such as genome replication or viral gene expression. For example, LL-37 has been shown to reduce respiratory syncytial virus replication even when added to already infected cells [34]. Finally, LL-37 tied up HIV-1 reverse transcriptase and protease through direct protein–protein interaction [35].

As a complement to direct antiviral activity, we attempt to determine whether the anti-HSV-1 effect of Hg-CATH and Pb-CATH4 could be related to immunomodulatory properties. The addition of AMPs to primary human keratinocytes did not result in a significant increase of mRNA expression of proinflammatory and antiviral genes, except for a moderate effect of Pb-CATH4 on CXCL8, TNF-α, and Viperin expression. Furthermore, a reduction of the innate antiviral response induced by HSV-1 was noticed in the presence of both peptides but the decrease of ISG mRNA expression might be explained by the strong reduction of viral replication observed in treated conditions. This suggests that Hg-CATH and Pb-CATH4 reduced HSV-1 replication independently of the potentiation of cellular antiviral defences.

Finally, our results showed that identically to what had been shown for BMAP-28, and LL-37, Hg-CATH and Pb-CATH4 do not affect infectivity of viral particles and therefore have no direct virucidal effects on HSV-1 [19,24]. Interestingly, all four peptides contain the A(A/N)–P–P–A(A/N) motif in their sequence. In contrast, indolicidin, a bovine cathelicidin, strongly inhibited HSV-1 infection by directly inactivating infectious viral particles [23,25]. The amino acid sequence and structure of indolicidin are quite different from those of the other anti-HSV-1 cathelicidins (BMAP-28, LL-37, Hg-CATH, and Pb-CATH4), suggesting a possible relationship between sequence homology of cathelicidins and their antiviral effects.

To conclude, in addition to their already described activity against Gram-negative bacteria, this work highlights, for the first time, the antiviral effect of Hg-CATH and Pb-CATH4 on HSV-1 [21,22]. Further investigations remain needed to decipher their mechanism(s) of action during the viral replication cycle. Owing to their abilities to inhibit viral replication and the production of infectious viruses by primary human keratinocytes, these AMPs may represent potential candidates for the development of innovative therapies against HSV infections.

## 4. Materials and Methods

### 4.1. Peptides

△Hg-CATH (XP_004834996.1) and △Pb-CATH4 (XP_007445036.2) identified in silico from the genome of *Heterocephalus glaber* and *Python bivittatus*, respectively, were synthetised by solid-phase peptide synthesis and purified by high-performance liquid chromatography (Figure 1) [21,22]. The 3D structure of Hg-CATH and Pb-CATH4 peptides was predicted using the LOMET server [36], and they were then energy minimised using GROMACS (GROningen MAchine for Chemical Simulations) [37]. Visualisation of their structures was performed using Pymol [38]. The sequence-dependent peptide characteristics of the peptides, including length, hydrophobicity, amphipathicity, and net charge, were reported in previous studies [21,22]. The helicities of the peptides were calculated using the hierarchical neural network secondary structure prediction method available from the Network Protein Sequence @nalysis server (http://npsa-pbil.ibcp.fr (accessed on 3 June 2021)) [23].

### 4.2. Virus Strain and Production

To produce the viral stock, monkey kidney epithelial cells, Vero (ATCC CCL-81), were infected at a multiplicity of infection (MOI) of 0.1 with an HSV-1 strain (ATCC VR260) for 3 days at 37 °C under 5% CO_2_ humidified atmosphere. Cell supernatants were centrifugated at 1000× *g* for 10 min. The clarified suspension was stored at −80 °C until use. The viral titre of 10^6.56^ TCID50/mL was determined by Kärber’s method using endpoint dilution assay.

### 4.3. Isolation and Culture of Normal Human Epidermal Keratinocytes from Skin Samples

The Ethics Committee of the Poitiers Hospital approved the use of human skin samples for research studies. After the provision of fully informed consent, normal abdominal or breast skin was obtained from patients undergoing plastic surgery. Primary human keratinocyte isolation and culture were carried out as previously described [39].

### 4.4. Cell Viability Assay

Primary keratinocyte viability was determined following incubation for 24 h with 5, 10, 20, and 40 µg/mL of Hg-CATH and Pb-CATH4, using the cell proliferation kit II (XTT, Roche, Basel, Switzerland), as previously described [19]. To perform an LDH assay, keratinocytes were cultured in 24-well plates. Supernatants collected after 24 h of stimulation were mixed with 500 µL of phosphate buffer solution (PBS)–0.1% Triton, while keratinocyte monolayers were lysed with 1 mL of PBS–0.1% Triton and sonicated for 30 s. The LDH activity was measured using the Cobas^®^ (Roche) analyser. Viability was calculated, yielding the ratio between the LDH released in supernatants and the total LDH measured in both supernatants and cell lysates.

### 4.5. Evaluation of the Antiviral Effects of the AMPs

The antiviral properties of Hg-CATH and Pb-CATH4 were evaluated during infection kinetics of primary human keratinocytes, which were incubated for 1 h with 10 and 20 µg/mL of Hg-CATH or 10 µg/mL of Pb-CATH4. In addition, 10 µg/mL of the anti-herpetic drug ACV was used as a positive control. Then, cells were infected by HSV-1 at an MOI of 0.1 or mock-infected for 1 h in presence of peptides. After removal of cell culture supernatant, the cell monolayer was rinsed twice with 500 µL of PBS. Then, 500 µL of fresh medium containing the initial concentration of Hg-CATH or Pb-CATH4 were added for a 23 h incubation. At 24 h postinfection, 100 µL of cell culture supernatants were harvested for viral DNA extraction in NucliSENS lysis buffer (BioMerieux, Marcy-l’Etoile, France), and 400 µL were stored at −20 °C until the determination of viral titre of cell culture supernatants made, as described below. The cell monolayer was lysed with 600 µL of NucliSENS lysis buffer before nucleic acid extraction. For evaluation of the immunomodulatory effect of the AMPs on keratinocytes, cells were stimulated by the AMPs alone for 24 h.

### 4.6. Nucleic Acid Extraction

The total nucleic acids from cell lysates and cell culture supernatants were extracted on the MagNA Pure Compact System (Roche) according to the manufacturer’s recommendations. Nucleic acid concentration and sample purity were evaluated using the Nanodrop 2000 spectrophotometer (Thermo Fisher Scientific, Waltham, MA, USA).

### 4.7. Viral DNA Quantification by Real-Time Polymerase Chain Reaction (qPCR)

HSV-1 genome quantification in cell lysates and cell culture supernatants was performed by qPCR targeting the unit-long (UL) 30 gene, as previously described [19].

### 4.8. Transcriptomic Analysis of the Inflammatory and Antiviral Response in Keratinocytes

Transcriptomic analysis of the inflammatory and antiviral response in keratinocytes was performed as previously described [19] using a panel of primers indicated in Table 1.

### 4.9. Viral Titration

The virucidal properties of Hg-CATH and Pb-CATH4 were assessed by preincubating 10^4.3^ TCID50 of HSV-1 with 10 and 20 µg/mL of Hg-CATH and 10 µg/mL of Pb-CATH4 in a final volume of 200 µL for 1 h at 37 °C. The residual infectious titre of the virus suspension was then measured using endpoint dilution assay on Vero cells and compared to that of the untreated viral suspension.

## Figures and Tables

**Table 1:** Sequences of primers used for RT-qPCR.

Gene	Forward (5′ → 3′)	Reverse (5′ → 3′)
CXCL8	TTGCCAAGGAGTGCTAAAGAA	AACCCTCTGCACCCAGTTTT
CXCL10	AAGGATGGACCACACAGAGG	TGGAAGATGGGAAAGGTGAG
G3PDH	GGCTCTCCAGAACATCATCCCTGC	GGGTGTCGCTGTTGAAGTCAGAGG
IFIT2	GCGTGAGAAGGTGAAGAGG	AATTTGGCAATGCAGGTAGG
MX1	ACCACAGAGGCTCTCAGCAT	ACCACAGAGGCTCTCAGCAT
Viperin	GGCAAGTTGGTGAGGTTCTG	ACCACCTCCTCAGCTTTTGA
RPS28	CCGTGTGCAGCCTATCAAG	CAAGCTCAGCGCAACCTC
TNF-α	TCACCCACACCATCAGCCGCATCG	GGGAAGGTTGGATGTTCGTCCTCC

## Data Availability

All data generated or analysed during this study are included in this article.

## Supplementary Materials

The following are available online at https://www.mdpi.com/article/10.3390/ph14080715/s1, Figure S1: Energy-minimised 3D structure of Hg-CATH and Pb-CATH4. The 3D structure of Hg-CATH (A) and Pb-CATH4 (B) were analysed by LOMETS server and visualised by Pymol after energy minimisation was conducted using Gromacs. Both peptides contain alpha-helical structures, Figure S2: Comparison of sequences and secondary structure predictions of five cathelicidins with anti-HSV-1 activity. The sequence-dependent peptide characteristics of the peptides were analysed such as length, hydrophobicity, amphipathicity, net charge, and helicity, Table S1: The sequence similarity of five cathelicidins with anti-HSV-1 activity.
