# Supplementary material for: Characterisation of Antiviral Activity of Cathelicidins from Naked Mole Rat and Python bivittatus on Human Herpes Simplex Virus 1
Source: Pharmaceuticals (Basel). 2021 Jul 24;14(8):715. doi: 10.3390/ph14080715 (PMC8398704; doi:10.3390/ph14080715)
Supplement: Supplementary file 1 [file pharmaceuticals-14-00715-s001.zip › pharmaceuticals-1292198-SI.pdf]

Supplementary material

(A) (B)

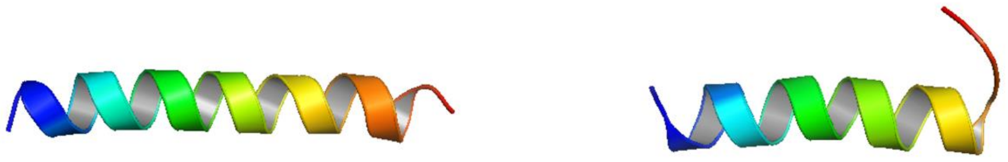

Figure S1: **Energy-minimized 3D structure of Hg-CATH and Pb-CATH4**. 3D structure of Hg-CATH (A) and Pb-CATH4 (B) were analyzed by LOMETS server and visualized by Pymol after energy minimization was conducted using Gromacs. Both peptides contain alpha-helical structure.

(A)

Peptides	Sequences and secondary structure predictions of mammalian cathelicidins with anti-HSV-1 activity																																			
Hg-CATH	S	K	F	F	R	K	A	R	K	K	L	G	K	G	L	Q	K	I	K	N	V	L	R	K	Y												
	c	h	h	h	h	h	h	h	h	h	h	h	h	h	h	h	h	h	h	h	h	h	h	h	c												
Pb-CATH4	T	R	S	R	W	R	R	F	I	R	G	A	G	R	F	A	R	R	Y	G	W	R	I	A													
	c	h	h	h	h	h	h	h	h	h	h	h	h	h	h	h	h	h	h	c	c	c	c	c													
LL-37	L	L	G	D	F	F	R	K	S	K	E	K	I	G	K	E	F	K	R	I	V	Q	R	I	K	D	F	L	R	N	L	V	P	R	T	E	S
	c	c	h	h	h	h	h	h	h	h	h	h	h	h	h	h	h	h	h	h	h	h	h	h	h	h	h	h	h	h	h	c	c	c	c	c	c
BMAP-28	G	G	L	R	S	L	G	R	K	I	L	R	A	W	K	K	Y	G	P	I	I	V	P	I	I	R	I	G									
	c	c	h	h	h	h	h	h	h	h	h	h	h	h	h	h	c	c	c	e	e	e	e	e	e	e	c	c									
Indolicidin	I	L	P	W	K	W	P	W	W	P	W	R	R																								
	c	c	c	c	c	c	c	c	c	c	c	c	c																								

h (alpha-helix) e (extended strand) c (random coil)

(B)

Peptide	Net charge (+)	Alpha helix (%)	Extended strand (%)	Random coil (%)
Hg-CATH	11	92	0	8
Pb-CATH4	9	75	0	25
LL-37	6	73	0	27
BMAP-28	8	50	25	25
Indolicidin	4	0	0	100

Figure S2: **Comparison of sequences and secondary structure predictions of five cathelicidins with anti-HSV-1 activity.** The sequence-dependent peptide characteristics of the peptides were analysed such as length, hydrophobicity, amphipathicity, net charge and helicity.

Table S1 : The sequence similarity of five cathelicidins with anti-HSV-1 activity.

	Hg-CATH	Pb-CATH4	LL-37	BMAP-28	Indolicidin
**Hg-CATH**	100	20	32	17	0
**Pb-CATH4**	-	100	14	25	12
**LL-37**	-	-	100	16	5
**BMAP-28**	-	-	-	100	10

Note. Global alignment analysis was performed using National Center for Biotechnology Information Search database (NCBI).
